# Morphological and molecular-biological features of glioblastoma progression in tolerant and susceptible to hypoxia Wistar rats

**DOI:** 10.1038/s41598-023-39914-9

**Published:** 2023-08-04

**Authors:** D. Sh. Dzhalilova, N. A. Zolotova, V. A. Mkhitarov, A. M. Kosyreva, I. S. Tsvetkov, A. S. Khalansky, A. I. Alekseeva, T. H. Fatkhudinov, O. V. Makarova

**Affiliations:** 1 Avtsyn Research Institute of Human Morphology of Federal State Budgetary Scientific Institution “Petrovsky National Research Centre of Surgery”, 3 Tsyurupy Street, Moscow, Russia 117418; 2 Research Institute of Molecular and Cellular Medicine, RUDN University, 6 Miklukho-Maklaya St, Moscow, Russia 117198

**Keywords:** Tumour heterogeneity, Inflammation, Risk factors, Experimental models of disease

## Abstract

Hypoxia is a major pathogenetic factor in many cancers. Individual resistance to suboptimal oxygen availability is subject to broad variation and its possible role in tumorigenesis remains underexplored. This study aimed at specific characterization of glioblastoma progression in male tolerant and susceptible to hypoxia Wistar rats. Hypoxia resistance was assessed by gasping time measurement in an 11,500 m altitude-equivalent hypobaric decompression chamber. Based on the outcome, the animals were assigned to three groups termed ‘tolerant to hypoxia’ (n = 13), ‘normal’, and ‘susceptible to hypoxia’ (n = 24). The ‘normal’ group was excluded from subsequent experiments. One month later, the animals underwent inoculation with rat glioblastoma 101.8 followed by monitoring of survival, body weight dynamics and neurological symptoms. The animals were sacrificed on post-inoculation days 11 (subgroup 1) and 15 (subgroup 2). Relative vessels number, necrosis areas and Ki-67 index were assessed microscopically; tumor volumes were determined by 3D reconstruction from histological images; serum levels of HIF-1α, IL-1β, and TNFα were determined by ELISA. None of the tolerant to hypoxia animals died of the disease during observation period, *cf.* 85% survival on day 11 and 55% survival on day 15 in the susceptible group. On day 11, proliferative activity of the tumors in the tolerant animals was higher compared with the susceptible group. On day 15, proliferative activity, necrosis area and volume of the tumors in the tolerant to hypoxia animals were higher compared with the susceptible group. ELISA revealed no dynamics in TNFα levels, elevated levels of IL-1β in the susceptible animals on day 15 in comparison with day 11 and tolerant ones. Moreover, there were elevated levels of HIF-1α in the tolerant animals on day 15 in comparison with day 11. Thus, the proliferative activity of glioblastoma cells and the content of HIF-1α were higher in tolerant to hypoxia rats, but the mortality associated with the tumor process and IL-1β level in them were lower than in susceptible animals. Specific features of glioblastoma 101.8 progression in tolerant and susceptible to hypoxia rats, including survival, tumor growth rates and IL-1β level, can become the basis of new personalized approaches for cancer diseases treatment in accordance to individual hypoxia resistance.

## Introduction

Hypoxia is a major pathogenetic factor in many cancers including brain tumors^1–5^. Oxygen deprivation within a tumor mass has been correlated with aggressiveness, metastatic potential and therapy resistance^1,5,6^. Development of effective anti-cancer therapies requires precise mechanistic understanding of this relationship^4^.

Cell response to hypoxia is orchestrated by master-regulator transcription factor HIF, (‘hypoxia-inducible factor’)^1,7^, a heterodimer of oxygen-dependently expressed α-subunit (HIF-1α, HIF-2α, or HIF-3α) and constitutively expressed β-subunit^1,7,8^. HIF-1α is activated in response to tissue hypoxia. Some of its targets, notably *VEGF (Vascular Endothelial Growth Factor)*, *EPO (Erythropoietin)* and *IGF2 (Insulin Like Growth Factor 2)*, have been implicated in tumor-induced angiogenesis^1,9^.

High immunohistochemical positivity for HIF-1α has been observed in a variety of human cancers^10,11^, the signal concentrated around necrotic lesions^11^. A positive correlation of HIF-1α expression with the rates of tumor growth, vascularization and metastasis has been demonstrated both clinically and experimentally^1^. Increased rates of HIF-1α synthesis in colon, gastric, lung, skin, ovarian, pancreatic and prostate gland carcinomas have been shown to correlate with tumor proliferation rates, malignancy grade and therapy resistance, and, ultimately, with adverse outcomes^10,11^. The adverse prognostic significance of HIF-1α levels has been confirmed for ovarian epithelial cancer, hepatocellular carcinoma, small cell lung cancer and breast cancers^12–15^. On the contrary, in some tumors, e.g. in light-cell kidney tumors, HIF-1 stabilization suppresses the tumor growth^16^.

Hypoxia and HIF-1α activation are important factors of disease progression and survival in glioblastoma (GBM)^3,17–19^. HIF-1α modulates tumor cell metabolism through its dependent genes (*GLUT*s, *VEGF*, etc.) promoting adaptation of tumor cells to hypoxia and stimulating angiogenesis^18,19^. In GBMs, HIF-1α is densely expressed around developing blood vessels and its levels correlate with neovascularization^20^. Around necrotic foci in GBM, HIF-1α concentrates in tumor cell nuclei, whereas in low-grade gliomas (e.g. anaplastic astrocytoma) it is mostly found in cytoplasm^9,17,20–22^. High levels of HIF-1α in GBM have been associated with low survival^23^.

Morbidity and lethality of many cancers correlate with geographical altitude^24–27^. Overall cancer death rates tend to decrease with altitude^24–27^, although trends for specific tumor types may differ. For lymphomas, breast cancers and head-and-neck carcinomas the lethality at higher altitudes is lower, whereas for hepatocellular and cervical cancers such correlation is negligible^24,25^. For lung cancer, morbidity and lethality negatively correlate with altitude^27^, whereas for hereditary paraganglioma type I (a carotid body cancer) higher altitudes provide a crucial phenotypic modifier^28^. Living in highlands is a significant risk factor for gastric cancer: increased morbidities have been reported for mountainous populations in Spain, Iran, Equador, China and South America^29,30^. Primary brain cancers, including GBM, are especially rare in populations living at > 400 m altitudes compared with other geographical areas^31^.

The observed decrease in cancer morbidity/lethality at higher altitudes may be mechanistically linked with adaptations to hypoxic environments. People living at lower atmospheric pressures present with higher hemoglobin content of the blood and express higher levels of HIF which protect them from hypoxia^32,33^. Such mechanisms often evolve locally: Tibetan, Andean and Ethiopian populations, each living at > 3500 m altitudes, exhibit different adaptation strategies^34,35^. Andeans have lower blood oxygen saturation but increased hemoglobin levels compared to Tibetan and Ethiopian highlanders^36,37^. Moreover, hemoglobin content, oxygen saturation and arterial oxygen levels in the Ethiopians (especially Amhara ethnic group) are similar to those of healthy individuals living at sea level^38,39^. In general, people living at > 2500 m altitudes are better fit to hypoxic conditions compared to average humans. Remarkable tolerance to oxygen deprivation among Nepalese living at 3570 m altitude is accompanied by high individual variation in hemodynamic parameters and hemoglobin content^40^ explained by highly variable genetic landscapes ensuring the adaptation^35^.

Humans and laboratory animals have different patterns of hypoxia resistance, varying broadly within species, strains and populations^41–46^. Experimental studies show that rodents with high and low hypoxia resistance differ by multiple parameters including HIF-1α and VEGF expression levels^41,42,45^. Under normal ambient pressures, susceptible to hypoxia rats express significantly higher neocortical levels of HIF-1α, which parallels the elevated baseline serum levels of HIF-1α in humans prone to high-altitude pulmonary edema^47^. Individual differences in HIF-1α expression levels may contribute to various diseases including cancers. As demonstrated by us previously, tolerant and susceptible to hypoxia animals differ by severity of systemic inflammatory response: susceptible rats develop a more severe inflammatory response accompanied by higher expression of HIF-1α^45^.

Life-long risks of cancer constitute 6.9% for lungs, 1.08% for the thyroid, 0.6% for the brain, 0.003% for pelvic bones and 0.00072% for laryngeal cartilage^48,49^. One of the hypotheses explaining the differences in the probability of malignant neoplasm at certain site within the body is based on peculiarities of the stem cell divisions intensity^50^. However, hypoxia contributes to mutations and heterogeneity of tumors and therefore it can also play an important role in the tumor development processes^3^. Experimental models reveal some hypoxia-related patterns, notably higher rates of melanoma B16 progression in susceptible to hypoxia mice^51^, but there is no data referring brain tumors. According to the literature, the brain and internal organs differ in their resistance to lack of oxygen. Meanwhile, brain structures, especially cerebral cortex and cerebellum, are known to be extremely sensitive to oxygen deprivation^52,53^. In this study we explored specific features of GBM progression in rats with high and low individual resistance to hypoxia.

## Methods

### Experimental animals

The study enrolled adult male Wistar rats (3 months old, body weight 220–250 g, n = 60) and was approved by Bioethics Committee at the Avtsyn Research Institute of Human Morphology (Protocol No. 21, March 29, 2019). All efforts were made to decrease suffering and possible stress for the animals and performed in accordance with the Directive 2010/63/EU of the European Parliament and of the Council of 22 September 2010 on the protection of animals used for scientific purposes. The reporting in the manuscript follows the ARRIVE guidelines. We used the method of blind research at all stages.

### Hypoxia resistance test

Hypoxia resistance was assessed through physiological response to oxygen deprivation using decompression chamber test as described previously^41–46,54,55^. The animals were exposed, one at a time, to simulated hypobaric hypoxia equivalent to 11,500 m altitude (180 mmHg) using a mercury barometer-coupled decompression chamber. Time length till the first sign of characteristic hyperventilatory response (‘gasping time’) was recorded using an electronic stopwatch. Based on gasping time, the animals were assigned to three groups: ‘susceptible’ (< 80 s, n = 24), ‘normal’ (80–240 s, n = 23) and ‘tolerant’ (> 240 s, n = 13). The ‘normal’ group was excluded from subsequent experiments. All rats survived the decompression chamber test and resumed their normal activity without signs of trauma. Test time was between 08.30 am to 12.30 pm and testing order was randomized daily. For each animal, different investigators were involved as follows: the first investigator determined the resistance to hypoxia. This investigator was the only person aware of the group allocation. The second investigator was responsible for the anesthetic procedure and tumor inoculation, whereas other investigators performed the rest analysis. All experimental procedures were performed by different researchers and they were not aware about the sample belonging to the specific group.

### Tumor inoculation

The orthotopic rat glioblastoma (GBM) 101.8 has been validated as a reliable and reproducible brain tumor model in studies on anti-tumor efficacy of nanoparticle-based doxorubicin formulations^56–61^. The model is characterized by rapid proliferation and invasive growth of tumor similar to human grade IV GBM^60,62^.

One month after hypobaric tests, the animals underwent intracranial inoculation of GBM 101.8 (~ 10^6^ cells) as described previously^57^. The animals were anaesthetized with 100 mg/kg ketamine and 10 mg/kg xylazine intraperitoneally. Parietal skin surface was treated with antiseptics and a 10 mm long longitudinal incision was made to the right of the midline. The right parietal bone was drilled with a dental bur 2 mm laterally from the sagittal suture and 2 mm caudally from the coronal suture, to get a hole 2 mm in diameter. The tumor material was injected to a 4 mm depth from the bone, corresponding to striatum area. Surgical glue (Turbo 2000 Kleber Universal, Boldt Co, Wermelskirchen, Germany) was used to close the scalp incision.

According to^62^ after 8, 10, 12 and 14 days of GBM inoculation all of the animals revealed tumors. On day 11 animals began to lose body weight, which indicated the progression of the tumor. On days 14–15 the maximum number of animals developed tumors of sufficient size to compare their volumes^57,60,63^. To trace the differences in the dynamics of tumor development in animals with different resistance to hypoxia, were suggested two terms. Experimental animals with an inoculated tumor were randomly divided into groups. The animals were sacrificed on post-inoculation day 11 (subgroup 1) or 15 (subgroup 2) with 15 mg/kg zoletil (Virbac Sante Animale, France) intramuscularly.

### Survival, symptoms and body weight monitoring

According to the literature, GBM 101.8 tumors start to manifest with characteristic neurological symptoms on post-inoculation day 14–18^57,60,63^. In our setting, no symptoms were observed by day 11, but in some animals the body weight decreased. By day 15, the animals developed lethargy, decreased locomotor activity, ruffled fur, paresis, and limb paralysis. The animals were weighted immediately before tumor inoculation on day 0 and subsequently on post-inoculation day 10 (survivors in subgroups 1 and 2) and post-inoculation day 14 (survivors in subgroup 2).

### Biological sample collection and processing

The blood was collected from jugular veins and after clotting centrifuged at 1500 g for 20 min; the serum was stored for ≤ 2 months − 70 °C. Brain tissues were fixed in 10% neutral formalin for 48 h and paraffin-embedded by standard procedure. Serial 4–5 μm thick histological sections were stained with H&E (BioVitrum, Saint Petersburg, Russia).

### Morphology and morphometry

Histological evaluation of tumors was randomized and blinded. Vascular densities were assessed microscopically by counting. Necrotic lesions were measured in μm^2^ over the entire tumor area in a section using ImageScope M software interactively with a Leica DFC290 camera.

### 3D reconstruction of tumors

Morphometric study used H&E stained stepped-serial frontal sections of the brain. Digital images were obtained with slide scanner and processed in Image-Pro Premiere 3D software (Media Cybernetics); the reconstruction involved 16–65 sections per tumor. Voxel size (Vx) was calculated using the Vx = X × Y × Z formula, with X × Y for linear size of tumor area in a section (mm^2^) and Z for voxel depth (section thickness + distance to the next section, mm)^64^.

### Immunohistochemistry

Immunohistochemical detection of Ki-67 was carried out by sandwich/fluorescence method. Brain sections were deparaffinized, demasked in citrate buffer pH 6.0 with 0.5% Tween-20 at 100 °C and blocked in phosphate-buffered saline with 0.1% bovine serum albumin at room temperature before exposure to antibodies (primary: Rabbit Polyclonal Anti-Ki67 Antibody, ab15580, Abcam, UK; secondary: Goat Anti-Rabbit IgG H&L Alexa Fluor 488 ab150077, Abcam). The nuclei were counterstained with DAPI (Sigma-Aldrich, USA). Digital images of peripheral, necrosis- and hemorrhage-free tumor regions (three fields of view at 200 × magnification) were captured using Zeiss Axioplan 2 fluorescence microscope in two channels with 488 nm and 358 nm excitation. The channels were merged in ImageJ software; Ki-67 index was determined as a percentage of Ki-67 positive cells among total tumor cell counts.

### ELISA

Serum levels of specific proteins were measured using reagent kits by Cloud-Clone Corp., USA, (IL-1β, TNFα) and FineTest, China, (HIF-1α) according to manufacturers’ protocols. The color reaction development was assessed in ANTHOS 2010 microplate analyzer (Anthos Labtec Instruments, Austria).

### Statistics

The analysis (Statistica 8.0, StatSoft) used nonparametric Mann–Whitney U test and multiple comparison procedures; each set of variables included ≥ 5 measurements. Multiple comparisons used Kruskal–Wallis test followed (at *p* < 0.05 only) by Dunn’s post-hoc test to validate the differences for each pair. Graphical representation used box-and-whisker plots of median, upper-lower quartile and upper-lower extreme values. Differences were considered significant at *p* < 0.05.

## Results

### Survival and body weight dynamics

**Figure 1:**
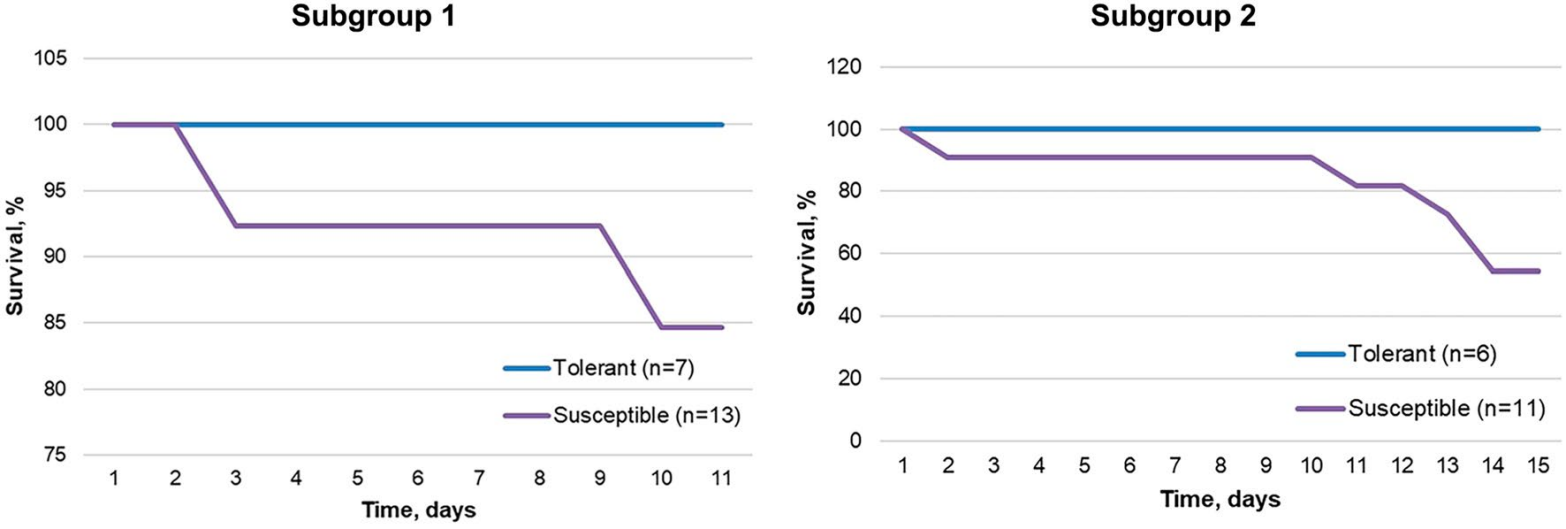
Survival curves for tolerant and susceptible to hypoxia rats, inoculated with GBM 101.8 on day 0.

None of the tolerant to hypoxia rats died until the end of observation, with entire subgroups 1 (n = 7) and 2 (n = 6) alive by days 11 and 15, respectively. In the susceptible to hypoxia group, 85% (11/13) of subgroup 1 survived by day 11 and 55% (6/11) of subgroup 2 survived by day 15. Most of the deaths occurred on day 9–14 post-inoculation (Fig. 1). In subgroup 1 animals, the body weight did not change during the experiment and not differ in tolerant and susceptible to hypoxia animals (Table 1). In subgroup 2 animals, the body weight also did not change throughout the experiment. However, in susceptible to hypoxia rats, a decrease in body weight by 7% by the end of the experiment was noticeable, while in tolerant rats it increased by 4%. At the same time, on post-inoculation day 10, the body weight of subgroup 2 susceptible to hypoxia rats was significantly lower in comparison to tolerant animals.

**Table 1:** Body weight dynamics in tolerant and susceptible to hypoxia rats, inoculated with GBM 101.8 on day 0.

Body weight, g	Tolerant (n = 7)	Susceptible (n = 13)	*p*
Subgroup 1			
Day 0	315.0 (300.0–400.0)	340.0 (340.0–350.0)	0.50
Day 10	310.0 (255.0–400.0)	348.0 (320.0–371.0)	0.41
	0.70	0.84	
Subgroup 2			
Day 0	350.0 (345.0–350.0)^1^	310.0 (297.0–344.0)^2^	0.17
Day 10	370.0 (360.0–373.0)^3^	299.0 (284.0–327.0)^4^	**0.037**
Day 14	365.0 (327.0–370.0)^5^	290.0 (263.0–320.0)^6^	0.16
	*p*^1–3^ = 0.29	*p*^2–4^ = 0.99	
	*p*^1–5^ = 0.99	*p*^2–6^ = 0.35	
	*p*^3–5^ = 0.99	*p*^4–6^ = 0.99	

Me (IQR); *p*—statistically significant differences, Kruskal–Wallis method with Dunn’s post-hoc test and Mann–Whitney test for tolerant and susceptible to hypoxia animals comparison.

Significant values are in bold.

### Morphological study

**Figure 2:**
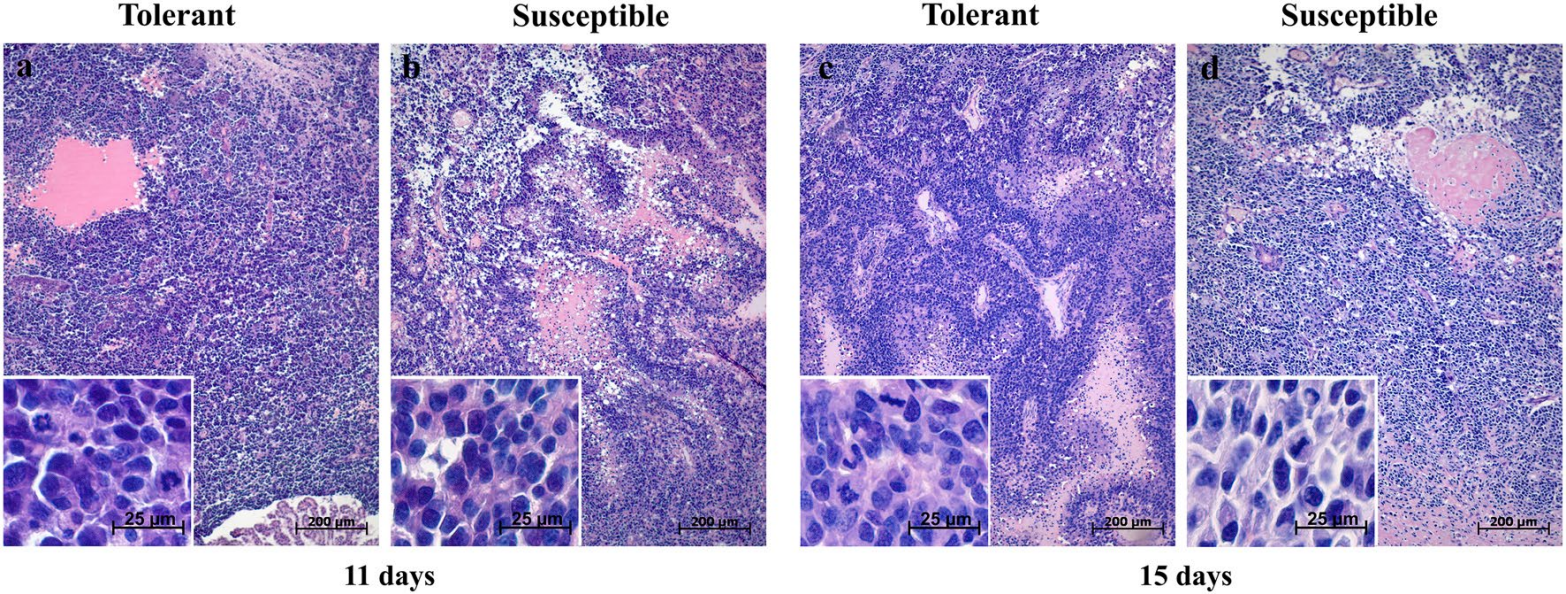
Morphological characterization of GBM 101.8 brain tumors in tolerant (**a**, **c**) and susceptible (**b**, **d**) to hypoxia rats on post-inoculation days 11 and 15. (**a**) tumor with atypical cell morphology sprouting the lateral ventricle wall; and hemorrhage foci; (**b**, **c**) tumors with palisade arrangement of atypical cells and massive necrosis; (**d**) tumor with atypical cell morphology and hemorrhage. The inserts show tumor cell polymorphism and atypia. Staining—H&E.

On post-inoculation day 11, both groups had small brain tumors composed of polymorphic atypical cells with high N/C ratio (Fig. 2). Histological examination revealed mitotic figures, small necrotic/hemorrhagic foci and signs of perivascular/perineuronal infiltration by tumor cells at the periphery. Blood vessels were visualized as thin-walled slit-like cavities and focal endothelial proliferation was noted.

On post-inoculation day 15, both groups had brain tumors of high cellularity with pronounced tumor cell atypia (Fig. 2). The cells contained hyperchromatic nuclei and were polymorphic in size and shape. Mitotically active and dying cells were distinguished. The tumors contained small hemorrhages and numerous necrotic lesions surrounded by cells with poorly visualized contours, vacuolated cytoplasm and weakly basophilic homogeneous nuclei. All tumors in both groups were richly vascularized with characteristic rosettes of 3–5 vessels—thin-walled, homogeneous in size and shape, with focal endothelial proliferation. Surrounding brain tissues had signs of perivascular and pericellular edema with hypo- and hyperchromic neurons predominating.

### 3D reconstruction of tumors

In clinical practice, assessment of tumor growth involves ultrasound, computed tomography and magnetic resonance imaging data. For experimental settings, utility of these approaches is limited by high costs and rarity of special equipment, as well as the low resolution (2–3 mm)^65^; accordingly, the measurements are mainly done post-mortem. For spherical/ellipsoidal tumors, the volume can be approximated as a product of maximum dimensions of the node in three perpendicular projections. Still, many tumors have highly irregular shape and cannot be measured by this method. A common solution is based on computer-assisted morphometry in digitized histological images. The use of special software (3D Reconstructor®, 3D-DOCTOR, etc.) enables reliable reconstruction of the object from stepped-serial histological sections^66,67^. Image Pro Premiere 3D Media Cybernetics software also affords accurate 3D reconstruction of tumor size and shape for experimental settings^64,68,69^. In this work the reconstruction was carried out as described previously^64^ yielding graphic volumetric images of GBM 101.8 brain tumors in tolerant and susceptible to hypoxia rats, corresponding to post-inoculation days 11 and 15.

**Figure 3:**
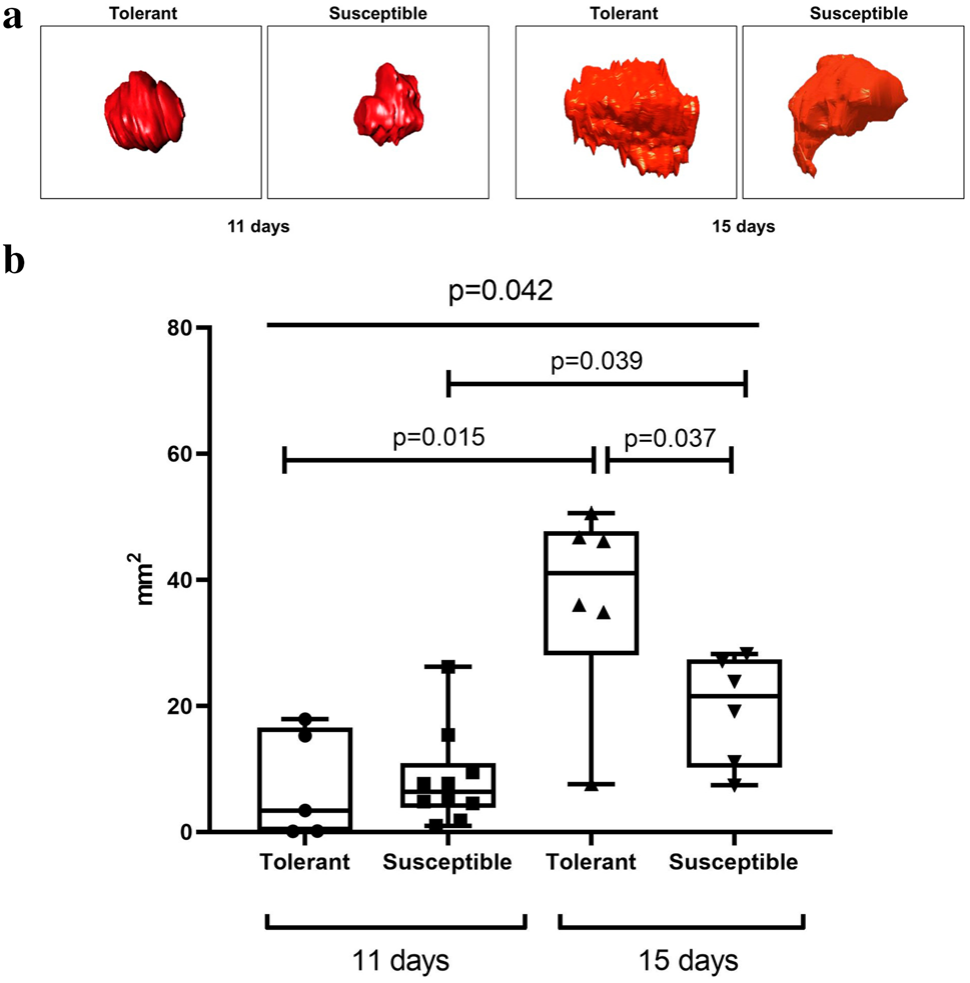
Representative 3D models (**a**) and estimated volume (**b**) of GBM 101.8 brain tumors in tolerant and susceptible to hypoxia rats on post-inoculation days 11 and 15. Me (IQR); *p*—statistically significant differences, Kruskal–Wallis method with Dunn's post-hoc test and Mann–Whitney test for tolerant and susceptible to hypoxia animals comparison. In all groups there were minimum 5 observations.

Size and shape of the tumors in both groups revealed considerable variation (Fig. 3a). The measurements revealed no between-the-group difference in tumor volume on post-inoculation day 11 (Fig. 3b) and significantly bigger tumors in the tolerant to hypoxia rats compared to the other group on post-inoculation day 15 (Fig. 3b).

### Tumor vessels number and necrosis areas

GBMs are highly vascularized tumors, which contributes to their aggressiveness^70^. Relative vessels number in brain tumors of tolerant and susceptible to hypoxia rats was similar at both time points of the observation (Table 2).

**Table 2:** Relative vessels number and necrosis areas in GBM 101.8 brain tumors of tolerant and susceptible to hypoxia rats on post-inoculation days 11 and 15.

Vessels, %	Tolerant	Susceptible	*p*
Day 11	14.0 (12.0–15.5)	12.8 (9.3–15.5)	0.54
Day 15	8.3 (6.5–11.5)	8.5 (7.0–11.0)	0.57
	0.08	0.10	

Necrosis area, µm^2^ × 10^3^	Tolerant	Susceptible	*p*
Day 11	328.0 (157.7–479.8)	90.7 (61.6–245.7)	0.17
Day 15	406.2 (360.4–511.2)	180.0 (136.5–192.0)	**0.01**
	0.56	0.36	

Me (IQR); *p*—statistically significant differences, Kruskal–Wallis method with Dunn’s post-hoc test and Mann–Whitney test for tolerant and susceptible to hypoxia animals comparison. In all groups there were minimum 5 observations.

Significant values are in bold.

An extra diagnostic marker for high-grade glioblastoma is the presence of vast necrotic areas in the brain. Necrosis is a major grading hallmark for gliomas associated with poor prognosis^71^. Tumor necrosis areas, similar in tolerant and susceptible to hypoxia rats on post-inoculation day 11, were significantly larger in the tolerant animals as compared to the other group on post-inoculation day 15 (Table 2).

### Tumor proliferation assay

**Figure 4:**
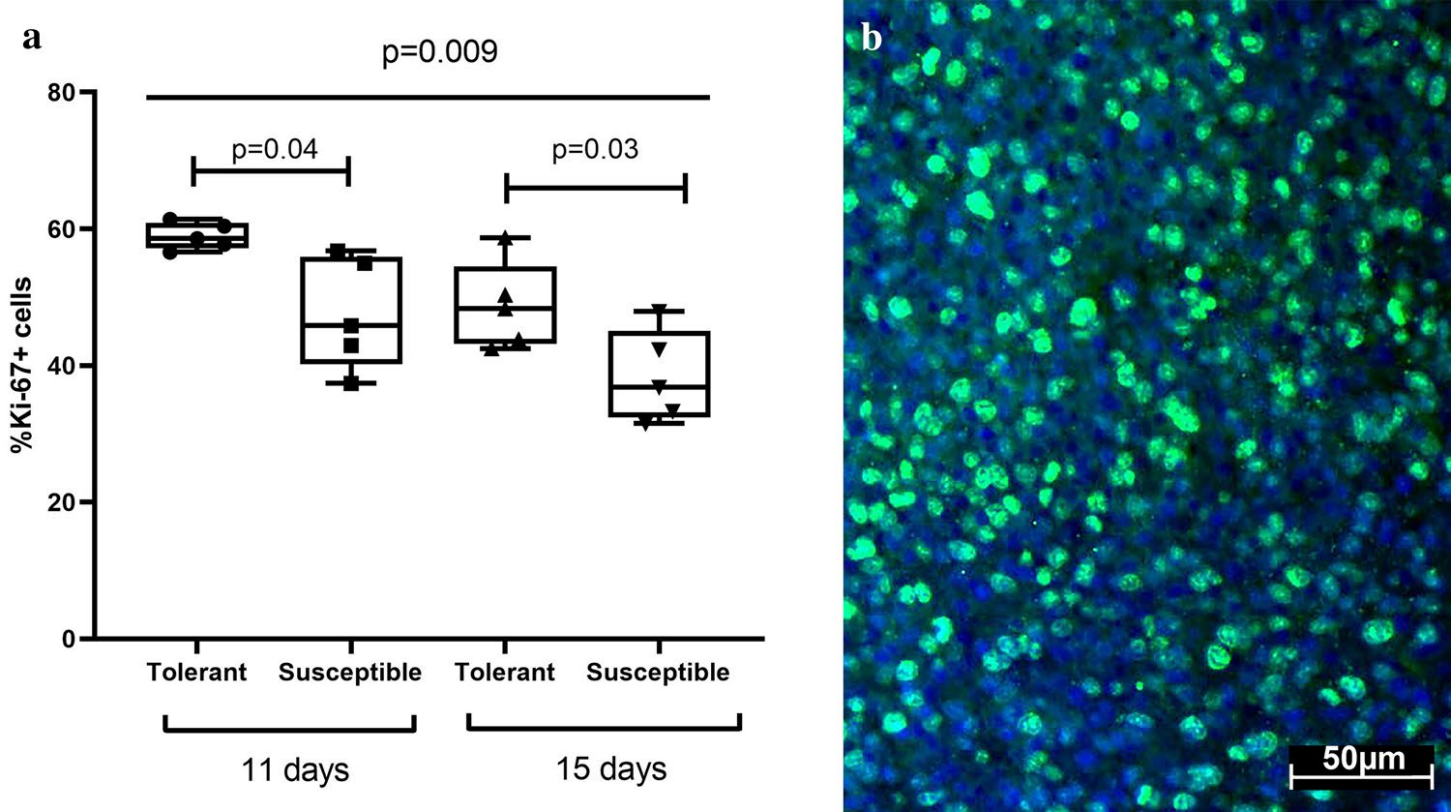
Proliferative activity of the tumors in tolerant and susceptible to hypoxia rats on post-inoculation days 11 and 15. (**a**) Ki-67 index boxplot; *p*—statistically significant differences, Kruskal–Wallis method with Dunn's post-hoc test and Mann–Whitney test for tolerant and susceptible to hypoxia animals comparison, %—Ki-67 + from total cells number. (**b**) representative immunostaining for Ki-67 (green) on post-inoculation day 11, cell nuclei counterstained with DAPI (blue). In all groups there were minimum 5 observations.

Proliferative activity of brain tumors was assessed using fluorescence immunostaining for Ki-67. Ki-67 is a nuclear antigen, which is expressed throughout all cell cycle phases except the G0-phase. The absence of Ki-67 in mitotically resting cells makes it a good proliferation marker ^72^. Ki-67 index was significantly higher in tolerant to hypoxia rats compared to the other group at both time points (post-inoculation days 11 and 15) and none of the groups revealed time-related dynamics for this parameter (Fig. 4).

### ELISA

**Figure 5:**
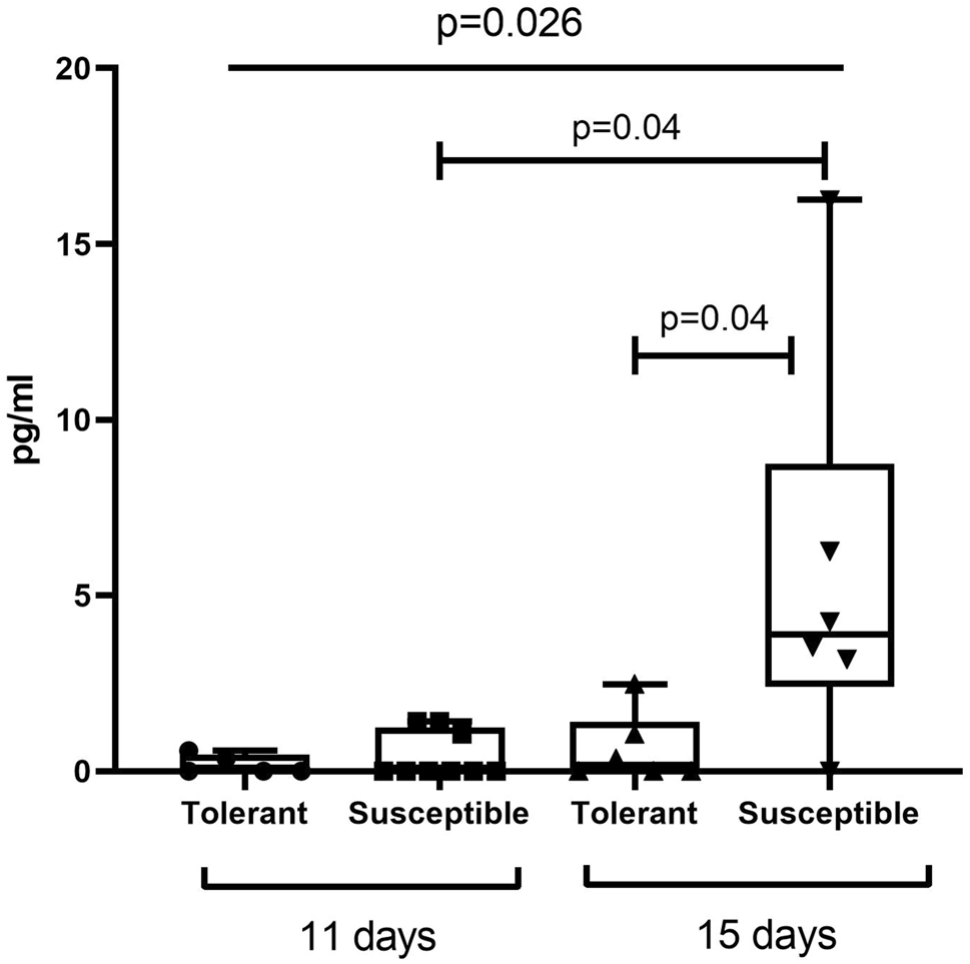
Serum levels of IL-1β in tolerant and susceptible to hypoxia rats on post-inoculation days 11 and 15; *p*—statistically significant differences, Kruskal–Wallis method with Dunn’s post-hoc test and Mann–Whitney test for tolerant and susceptible to hypoxia animals comparison. In all groups there were minimum 5 observations.

**Figure 6:**
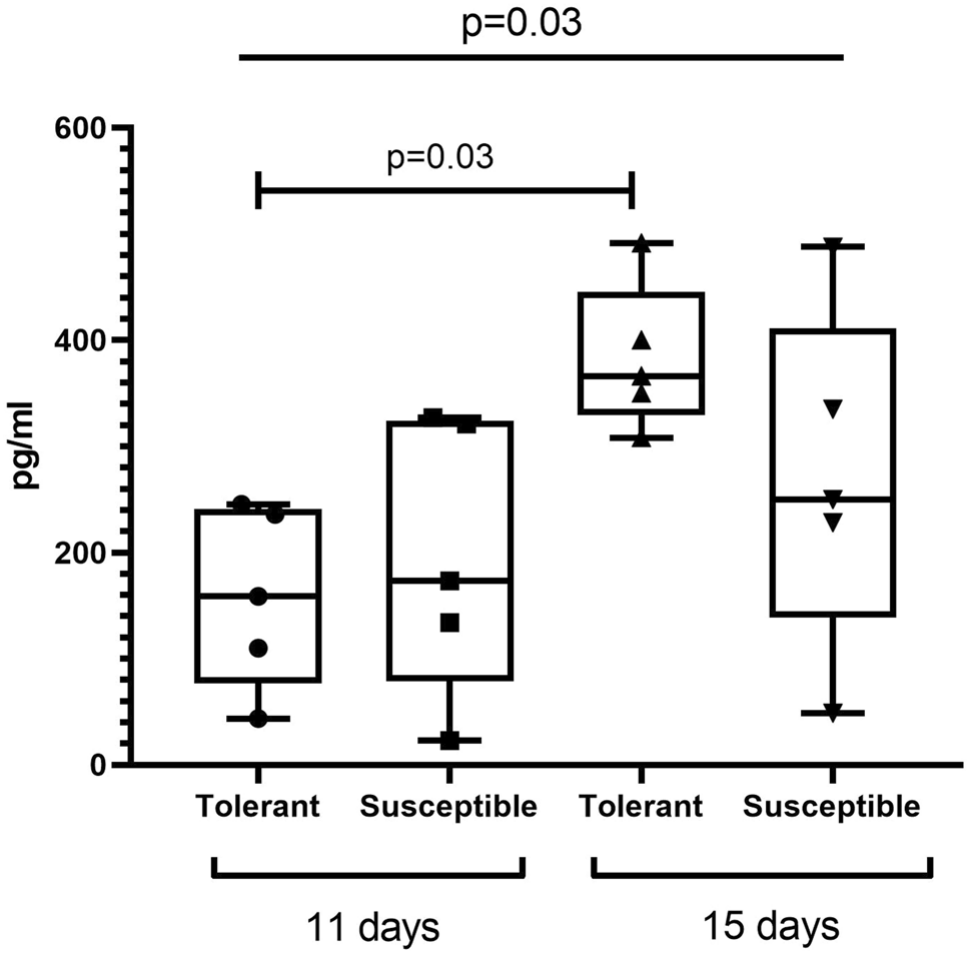
Serum levels of HIF-1α in tolerant and susceptible to hypoxia rats on post-inoculation days 11 and 15; *p*—statistically significant differences, Kruskal–Wallis method with Dunn’s post-hoc test and Mann–Whitney test for tolerant and susceptible to hypoxia animals comparison. In all groups there were minimum 5 observations.

The analysis revealed no differences in serum levels of TNFα between tolerant and susceptible to hypoxia rats, except a downward trend in this parameter for the tolerant rats on post-inoculation day 15 as compared to the other group (Table 3). Serum levels of IL-1β were significantly increased in the susceptible to hypoxia rats on post-inoculation day 15 compared to both the other group and the measurement on day 11 (Fig. 5). Serum levels of HIF-1α were similar between the groups at both time points, while showing a significant upward trend in tolerant to hypoxia group only (Fig. 6).

**Table 3:** Serum levels of TNFα in tolerant and susceptible to hypoxia rats on post-inoculation days 11 and 15.

pg/mL	Tolerant	Susceptible	*p*
TNFα			
Day 11	15.6 (0.2–31.2)	6.8 (0.1–42.5)	0.92
Day 15	0.98 (0.1–19.4)	5.9 (5.9–8.8)	0.27
*p*	0.81	0.93	

Me (IQR); *p*—statistically significant differences, Kruskal–Wallis method with Dunn’s post-hoc test and Mann–Whitney test for tolerant and susceptible to hypoxia animals comparison. In all groups there were minimum 5 observations.

Comparative characterization of GBM 101.8 progression in tolerant and susceptible to hypoxia rats is given in Table 4. The progression of glioblastoma in susceptible to hypoxia Wistar rats leads to the death of animals and is accompanied by an increase in the IL-1β level. At the same time, in tolerant to hypoxia rats, despite the large size of the tumor and the necrosis area, high tumor cells proliferative activity, an increase in HIF-1α level in blood serum on post-inoculation day 15, death of animals and the increase in the IL-1β level were not observed.

**Table 4:** A summary on GBM 101.8 progression in tolerant and susceptible to hypoxia rats.

Parameter	Tolerant, day 11 versus day 15	Susceptible, day 11 versus day 15	Dif. tolerant versus susceptible
Survival	No dynamics	↓	↑ in tolerant throughout observation
Body weight	No dynamics	No dynamics	none
Tumor volume	↑	↑	↑ in tolerant on day 15
Proliferative activity	No dynamics	No dynamics	↑ in tolerant on days 11 and 15
Vessels number	No dynamics	No dynamics	None
Necrosis area	No dynamics	No dynamics	↑ in tolerant on day 15
Serum TNFα	No dynamics	No dynamics	none
Serum IL-1β	No dynamics	↑	↓ in tolerant on day 15
Serum HIF-1α	↑	No dynamics	None

Arrows indicate statistically significant differences: ↑, increased; ↓, decreased.

## Discussion

Glioblastomas (GBMs) are extremely aggressive primary brain tumors^73,74^ with median survival 12–15 months post-diagnosis^75^. In this study we used a previously established rodent model of GBM to comparatively characterize disease progression in animals with different hypoxia resistance regarded as a trait. It is known that there are organisms resistant to hypoxia and the development of cancer, for example, naked mole rat and blind mole rat, which is substantiated due to a high level of protection against oxidative stress^76–79^. We have demonstrated for the first time that within the same species there are also differences in resistance to tumor progression. This can probably be due to differences in the functional state of mitochondria, antioxidant defense enzymes, and immune system cells, which is discussed in detail in^46^.

Survival, the ultimate tumor progression indicator, was significantly lower in susceptible to hypoxia animals. Under normal physiological conditions, tolerant to hypoxia rats have been shown to live 15% longer than susceptible^80^, which can be explained as follows—tolerance to hypoxia correlate of overall physical fitness, toxin inactivation capacity, locomotor potential, physiological stress endurance and adaptability, and probably also immunological plasticity of the body. Moreover, the tolerant to hypoxia animals respond to unfavorably low oxygen pressures by expressing higher levels of antioxidant protection enzymes and heat-shock proteins, while the susceptible to hypoxia animals enter oxidative stress^42,43^. According to^81,82^ in tolerant and susceptible to hypoxia animals there were revealed the structural differences of mitochondria, which play a pivotal role in oxygen sensing and free radical generation. The mitochondria of the cerebral cortex cells, liver and heart of tolerant and susceptible to hypoxia rats differ in both structural and basic functional parameters. In tolerant animals the cells of the cerebral cortex under normoxia were characterized by the high content of mitochondria with more densely packed cristae and dark matrix, large number of small mitochondria, and a higher concentration of mitochondrial enzymes such as Subunit A of Succinate Dehydrogenase (SDHA), Cytochrome b (Cyt b), Cytochrome C Oxidase Subunit I (COX1), and succinate versus mitochondria in susceptible rats. On the contrary, the number of mitochondrial cristae in the brain mitochondria of susceptible to hypoxia rats was less than in tolerant animals. Smaller mitochondria with a denser packing of cristae are functionally more active^83–85^, which is consistent with the higher basic functional activity of mitochondrial energy apparatus in the cerebral cortex in tolerant to hypoxia rats in comparison to susceptible rats. In tolerant to hypoxia rats the large number of small mitochondria in the cerebral cortex is an indicator of increased metabolic mitochondrial activity and higher intensity of oxidative phosphorylation in these rats in comparison to susceptible rats. Different intensities of oxidative phosphorylation in the cerebral cortex in rats with tolerant and susceptible hypoxia resistance was also demonstrated earlier^86,87^. Thus, phenotypic ultrastructural, functional and metabolic differences were observed between the mitochondria of cerebral cortex cells in animals with different hypoxia tolerance. They indicate greater activity of the respiratory chain in rats tolerant to hypoxia in comparison to susceptible rats. These differences also suggest that energy metabolism is a determining factor in individual tolerance to hypoxia. Mitochondria play an important role in cancer through macromolecular synthesis and energy production^88^. In tumor cells metabolic changes were detected, in particular, glycolysis was activated, the rate of which was higher than oxidative phosphorylation. Metabolic changes, glycolysis activation, and formation of the acidic environment facilitate cell proliferation and tumor progression^89^. Since tolerant rats demonstrated higher activity of the cells energy apparatus, it is likely that after inoculation, tumor cells get into favorable conditions and actively proliferate, which leads to a larger volume of tumors in tolerant animals. It is likely that effective protective mechanisms, including antioxidant defense enzymes and heat shock proteins, in tolerant to hypoxia animals contribute to their survival, despite the pronounced tumor progression.

The high proliferative activity of tumor cells determines its growth and size increase; however, there is no definite relationship between tumor size and animal lifespan. Predicting the course of the disease based on the proliferative potential of tumor cells is a complex issue and requires further research. Ki-67 proliferation marker is a nuclear protein expressed by many cancers^90^ and its expression has been related to specific aspects of tumor progression including invasion and angiogenesis^91^. For hepatocellular carcinoma and astrocytoma, Ki-67 index has been correlated with histological grade and lethality^92,93^. For GBM, prognostic value of Ki-67 index is controversial^94–97^, despite its low value as independent predictor^98^, Ki-67 has been identified as survival marker for reoperated cases in GBM^99^. These data are consistent with a recently reported positive correlation between Ki-67 index and overall survival in GBM^100^. In our experiments, tolerant to hypoxia rats showed higher Ki-67 index of tumors and better survival throughout the observation period.

Necrosis in GBM is a diagnostic hallmark correlating with tumor aggressiveness and fatal outcome^71,101^. Similarly with other rapidly growing solid tumors, GBMs are hypoxic, likely due to the rapid tumor expansion outstripping vascular supply^102^. Under these conditions, necrosis can be triggered by hypoxia and abrupt depletion of intracellular ATP without means for its replenishment through oxidative phosphorylation. Still, the nature of necrosis in GBM is not well-studied, but it is complex and is likely to involve multiple mechanisms, notably the neutrophil-triggered ferroptosis^103^. There is no straightforward relationship between necrotic lesions and tumor volume: small GBMs also develop necrosis^102,103^. Pseudopalisading necrosis and ‘chaotic’ angiogenesis are histological hallmarks of GBM^104^. In our experiments, none of the tolerant to hypoxia animals died by day 15 of observation, despite significantly bigger tumor volume and necrotic area (respectively, 1.9- and 2.3-fold compared with survivors in the other group, 45% of which were dead by this time point). Of note, relative volume of necrotic lesions and survival in GBM have been clinically correlated, with life span sharply reduced in patients having > 35% of tumor volume occupied by necrosis^101^. In our experiments, necrotic lesions constituted < 35% of tumor volume in both groups, which may explain the lack of correlation between necrosis and survival as assessed on day 15.

GBM progression is accompanied by oxygen deprivation at the site of tumorigenesis, leading to local activation of HIF-1, which further supports proliferation and angiogenesis in a positive feedback manner^19,73^. Apart from adaptation of tumor cells to low oxygen levels, HIF-1 has been implicated in specific aspects of GMB progression including immortalization, invasion and metastasis^18^. HIF-1 inhibition is considered a potential medication strategy for GBM expected to impact tumor dedifferentiation, angiogenesis and autophagy, prevent cytotoxicity resistance and improve survival^19,105^. According to our data, the necrosis area in tolerant to hypoxia rats was higher on post-inoculation day 15 in comparison to susceptible ones. Moreover, we observed significantly increased serum levels of HIF-1 in the tolerant to hypoxia group on post-inoculation day 15, consistently with previous reports on stronger HIF-1 activation in tolerant rats under conditions of acute hypoxic exposure^42,44^. Therefore, in tolerant to hypoxia rats, the progression of glioblastoma is accompanied by the development of necrosis, severe hypoxia, and HIF-1 activation. Nevertheless, despite the more pronounced HIF-1 activation, probably due to the effective defense systems and adaptation to hypoxia, the survival rate of tolerant to hypoxia rats is higher in comparison to susceptible ones. However, increased HIF serum levels in the tolerant rats on post-inoculation day 15 may be attributed simply to greater cell necrosis that favors the release of HIF protein in the bloodstream. Therefore, it is possible that elevated serum HIF levels do not reflect its activation.

Human GBMs are known to produce IL-1β in high amounts correlating with aggressiveness and adverse prognosis^106^. Cancer-associated systemic inflammation is a recognized phenomenon involving massive release of cytokines, small inflammatory molecules and immune cells into systemic circulation with dire immunological consequences^107^ including paraneoplastic neurodegeneration, autoimmune reactions^108–110^ and inflammatory conditions such as dermatomyositis^111^ and polymyositis^112^. Cancers also entail cardiovascular component with high risks of strokes, infarctions, cardiac failure and pulmonary thromboembolism, depending on patient’s age and stage/grade of the tumor^113^. Increased serum levels of TNFα and IL-1β in patients with brain tumors have been associated with adverse prognosis^114^. In this study, we observed elevated serum levels of IL-1β in the susceptible to hypoxia group on post-inoculation day 15 and no between-the-group differences for TNFα. Thus, in susceptible to hypoxia rats, cancer-associated systemic inflammation was more pronounced.

HIF-1 and IL-1β have been shown to reciprocally support expression of one another^115,116^. Hypoxia in pseudopalisading cells surrounding micronecrotic areas promotes infiltration by inflammatory cells further stimulating HIF-1 activation^116,117^ and such inflammatory foci within the tumor contribute to GBM progression^118^. Connection of HIF-1 with inflammation markers IL-1β and NF-κB provides important link between inflammation and tumorigenesis in GBM^116^. The  *IL1B* gene, a target of HIF-1, has been implicated in tumor growth, metastasis, invasiveness and angiogenesis^115,119^, partially by regulating other pro-inflammatory cytokine and growth factor secretion^120^. Importantly, IL-1β protein expression levels in clinical samples of glioma have been correlated with WHO grade^121^. As long as baseline HIF-1 expression levels in brain tissues of susceptible to hypoxia rats are higher^41,46^, it is possible that GBM 101.8 triggered systemic inflammatory response specifically in this group, boosting the early death rates. As shown by us previously, susceptible to hypoxia rats have more severe systemic inflammatory response with higher levels of IL-1β^45^.

One of the problems of biomedical research is the reproducibility of results. Phenotypic plasticity leads to variation in results, even if all animals are genetically identical^122,123^. Plastic responses of an organism with a specific genotype towards its local environment (i.e., the laboratory environment) may result in remarkably different results across replicate studies^124^. In addition to sex and age differences^125^, individual resistance to hypoxia should also be considered. The broad range of tumor volume in experimental GBM 101.8^126^ may reflect the variability of individual physiological traits of the host including hypoxia resistance. Individual hypoxia resistance is not taken into account in experimental investigations. B16 melanoma progression was previously demonstrated as more pronounced in susceptible to hypoxia mice^51^. Nevertheless, since each type of tumor has its own characteristics and depends differently on the lack of oxygen, our findings indicate that tumor progression patterns in GBM, including tumor volume, systemic inflammatory response and survival, depend on individual resistance of the body to environmentally inflicted hypoxia. A purely physiological explanation would be that animals qualified as ‘tolerant to hypoxia’ using the hypobaric chamber test are inherently more apt in capturing atmospheric oxygen during breathing and/or have higher capacity for its transportation, higher rates of oxygen exchange in peripheral tissues and energy metabolism, etc., and hence are generally better adapted in terms of oxygenation, which favors both the increase in tumor mass volume and host survival. In this regard, personalized accounting for hypoxia resistance as a trait in GBM may prove clinically relevant.

Our study has certain limitations. It was revealed that tolerant rats display better survival, but greater tumor volume than the susceptible ones. In perspective, this result may emphasize that it is not the lack of oxygen, but rather the individual resistance to hypoxia determines the tumor cells growth. This concept is rather novel, but more data is required to support it. In particular, it is necessary to identify specific mechanisms that provide better survival in tolerant animals. In our investigation, animals were transplanted with the same standard tumor, rat glioblastoma 101.8, while the reaction in animals with different resistance to hypoxia was different. It is likely that the reaction is largely due to the reactions of the microenvironment and the immune system, which counteract the growth of the tumor. According to our previous data, tolerant to hypoxia rats have more pronounced cellular and innate immunity, while humoral immunity is more pronounced in susceptible animals^127^. In addition, in susceptible to hypoxia animals, a more severe course of the systemic inflammatory response, acute and chronic ulcerative colitis were revealed^45,128,129^. Therefore, it is necessary to conduct additional studies on the tumor process features in animals with different resistance to hypoxia, which will allow to reveal mechanisms and to develop new personalized approaches to tumor therapy.

In conclusion, we have demonstrated for the first time that there are dependent from resistance to hypoxia differences in rats susceptibility to tumor progression. The progression of glioblastoma 101.8 in susceptible to hypoxia Wistar rats was characterized by high mortality and an increase in the IL-1β level. At the same time, in tolerant to hypoxia rats, despite the large size of tumor and the necrosis area, high tumor cells proliferative activity, an increase in HIF-1α level, death of animals and increase in the IL-1β level were not observed. Specific features of glioblastoma 101.8 progression in tolerant and susceptible to hypoxia rats, including survival, tumor growth rates and IL-1β level, can become the basis of new personalized approaches for cancer diseases treatment in accordance to individual hypoxia resistance.

## Data Availability

The data that support the findings of this study are available on request from the corresponding author.
